# First-Principles Molecular Dynamics Simulations on Water–Solid Interface Behavior of H_2_O-Based Atomic Layer Deposition of Zirconium Dioxide

**DOI:** 10.3390/nano12244362

**Published:** 2022-12-07

**Authors:** Rui Xu, Zhongchao Zhou, Yingying Wang, Hongping Xiao, Lina Xu, Yihong Ding, Xinhua Li, Aidong Li, Guoyong Fang

**Affiliations:** 1 Key Laboratory of Carbon Materials of Zhejiang Province, College of Chemistry and Materials Engineering, Wenzhou University, Wenzhou 325035, China; 2 National Laboratory of Solid State Microstructures, College of Engineering and Applied Sciences, Nanjing University, Nanjing 210093, China

**Keywords:** zirconium dioxide (ZrO_2_), H_2_O-based atomic layer deposition (H_2_O-based ALD), water–solid interface reaction, first-principles molecular dynamics (FPMD), density functional theory (DFT)

## Abstract

As an important inorganic material, zirconium dioxide (ZrO_2_) has a wide range of applications in the fields of microelectronics, coating, catalysis and energy. Due to its high dielectric constant and thermodynamic stability, ZrO_2_ can be used as dielectric material to replace traditional silicon dioxide. Currently, ZrO_2_ dielectric films can be prepared by atomic layer deposition (ALD) using water and zirconium precursors, namely H_2_O-based ALD. Through density functional theory (DFT) calculations and first-principles molecular dynamics (FPMD) simulations, the adsorption and dissociation of water molecule on the ZrO_2_ surface and the water–solid interface reaction were investigated. The results showed that the ZrO_2_ (111) surface has four Lewis acid active sites with different coordination environments for the adsorption and dissociation of water. The Zr atom on the surface can interacted with the O atom of the water molecule via the *p* orbital of the O atom and the *d* orbital of the Zr atom. The water molecules could be dissociated via the water–solid interface reaction of the first or second layer of water molecules with the ZrO_2_ (111) surface. These insights into the adsorption and dissociation of water and the water–solid interface reaction on the ZrO_2_ surface could also provide a reference for the water–solid interface behavior of metal oxides, such as H_2_O-based ALD.

## 1. Introduction

Zirconium dioxide (ZrO_2_) is an important inorganic material and has wide applications in the fields of microelectronics, coating, catalysis, energy storage and conversion [1,2,3,4,5,6]. With the further scaling-down of metal-oxide semiconductor field-effect transistor (MOSFET) devices, the traditional dielectric material, silicon dioxide (SiO_2_), fails to overcome the quantum mechanical tunneling effect in the devices [7,8,9,10,11,12,13]. Due to its high dielectric constant and thermodynamic stability, ZrO_2_ has been used as the dielectric material to replace SiO_2_. Currently, ZrO_2_ films can be prepared by physical vapor deposition (PVD), chemical vapor deposition (CVD) and atomic layer deposition (ALD) methods. Among these techniques, ALD has an obvious advantage for the gate dielectric ultrathin film for the nanodevices. ALD can accurately control the composition and thickness of the deposited thin films at the atomic level by alternately pumping different precursors into the reactor. It can utilize the self-limiting and self-saturation features of surface reactions, which result in the advantages of excellent three-dimensional conformity, large-area uniformity and precise thickness control of thin films [14,15,16,17,18]. Usually, water can be used as an oxygen source for the ALD preparation of metal oxides, such as Al_2_O_3_, TiO_2_, ZrO_2_ and HfO_2_. Thus, the water–solid interface is an important scientific issue for H_2_O-based ALD of metal oxides. Meanwhile, the water–solid interface also performs very important roles in various fields of nature including microelectronics, coating and heterogeneous catalysis [19,20,21,22].

ZrO_2_ has three crystalline phases, namely, common monoclinic, tetragonal and cubic crystals. The adsorption behavior of water molecules on the surface of ZrO_2_, such as cubic ZrO_2_ (111) and (110) and monoclinic ZrO_2_ (001), ($\bar{1}$01) and ($\bar{1}$11), have been studied [23,24,25,26,27,28,29,30]. For example, Korhonen et al. studied the adsorption of water on the most stable ($\bar{1}$01) and ($\bar{1}$11) surfaces of monoclinic ZrO_2_ using density functional theory (DFT) and infrared spectroscopy [26,27]. Xia et al. examined the hydration of the m-ZrO_2_ ($\bar{1}$11) surface using ab initio molecular dynamics simulations [29]. By obtaining the phase diagrams of surface dehydration, the representative partially hydrated m-ZrO_2_ ($\bar{1}$11) surfaces at various temperatures are illustrated. The Fourier transform infrared (FTIR) spectroscopy has demonstrated that the m-ZrO_2_ surfaces have amounts of acidic and basic hydroxyl groups and undissociated coordinated water molecules [30]. Herein, we focused on the adsorption and dissociation of water molecules on the relatively stable surface of monoclinic ZrO_2_ (111) and its water–solid interface reactions using DFT calculations and first-principles molecular dynamics (FPMD) simulations. The structure and density of states (DOS) before and after adsorption and dissociation of water are also investigated. The results show that the Zr atom of the ZrO_2_ (111) surface is the active site for the adsorption and dissociation of water. The water molecules close to the surface will be strongly adsorbed on the substrate and form Zr–O bonding sites. Some water molecules will be directly dissociated and form O–H bonds with the O atoms on the substrate surface. The dissociation of water can also occur via a proton exchange reaction between two water molecules on the surface of the monoclinic ZrO_2_ (111) surface. These modeling and insights into the water–solid interface reaction of ZrO_2_ via FPMD simulations at the atomic level can provide theoretical references for water–solid interface studies of metal oxides, such as H_2_O-based ALD.

## 2. Computational Details

First, the monoclinic ZrO_2_ crystal (space group *P*_21/c_) was optimized. The optimized lattice parameters are 5.16, 5.23 and 5.35 Å, respectively, which are in accordance with the experimental values of 5.15, 5.21 and 5.31 Å [31]. The average length of the Zr–O bond is 2.295 Å. To model the surface, a monoclinic ZrO_2_ (111) (1 × 2) surface with 13.37 × 7.40 Å^2^ was adopted. The model includes two, three, four and five layers of ZrO_2_. For the structural optimization, the atoms of the lowest layer of the surface were fixed. To ensure that there is enough space to adsorb water molecules and avoid the influence between two interfaces, the vacuum between the two surfaces was set to 20 Å. For the FPMD simulations, three layers of O–Zr–O including 72 atoms were used. At the same time, 55 water molecules were placed in the 20 Å vacuum.

All geometric optimizations were performed using DFT calculations based on the generalized gradient approximation with the Perdew–Burke–Ernzerhof functional and implemented in the Vienna Ab initio Simulation Package (VASP 6.2) [32]. The valence electronic states were expanded based on plane waves with the core–valence interaction represented using the projector augmented plane wave approach [33,34]. An energy cutoff of 520 eV was used. A Γ-centered k-mesh of 7 × 6 × 6 was used for the calculations of the crystal. A Γ-centered k-mesh of 2 × 4 × 1 was used for the calculations of the surface. The convergence criteria for total energies and the forces for ionic relaxation were 1 × 10^−5^ eV and 0.02 eV/Å, respectively. For the FPMD simulations, the *NVT* ensemble was used. The temperature was controlled at 373 K using a Nose-Hoover thermostat. The time step was set to 1.0 fs. The total time of the FPMD simulation was 15 ps.

## 3. Results and Discussion

### 3.1. Surface Model

**Figure 1:**
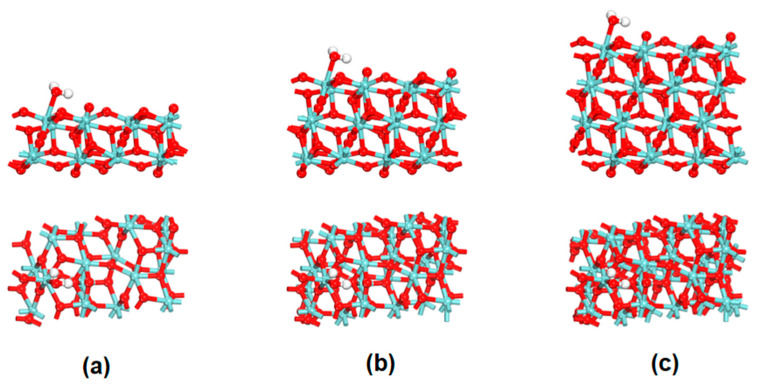
Structure of a water molecule adsorbed on two-layer (**a**), three-layer (**b**) and four-layer (**c**) models of the monoclinic ZrO_2_ (111) surface.

Surface model tests were performed using two, three and four layers of ZrO_2_ surface, shown in Figure 1. The water molecule was adsorbed on the same site of the ZrO_2_ surface models. The structural parameters and the adsorption energy are shown in Table 1. Here, the adsorption energy can be defined as:

*E*_ads_ = *E*_(substrate + adsorbate)_ − *E*_substrate_ − *E*_adsorbate_ (1)

where *E*_(substrate + adsorbate)_, *E*_substrate_ and *E*_adsorbate_ represent the energies of the system, surface and adsorbate, respectively. The results show that there is almost no difference in the adsorption configuration of the water molecule on the surface models of two, three and four layers. The three adsorption energies are about −18.5 kcal/mol. Due to Zr atoms on the surface are strong Lewis acid sites, water can be strongly adsorbed on the ZrO_2_ surface, indicating that this reaction is driven by strong adsorption of adsorbates at strong Lewis acid sites. Meanwhile, all of this indicates that after the surface model reaches a certain thickness, the increase in the substrate thickness has little effect on the configuration and adsorption energy of water molecules on the surface. Therefore, to balance the computational accuracy and cost, the three-layer model of the ZrO_2_ surface was adopted for the following calculations.

### 3.2. Adsorption and Dissociation of a Water Molecule on the ZrO_2_ (111) Surface

**Figure 2:**
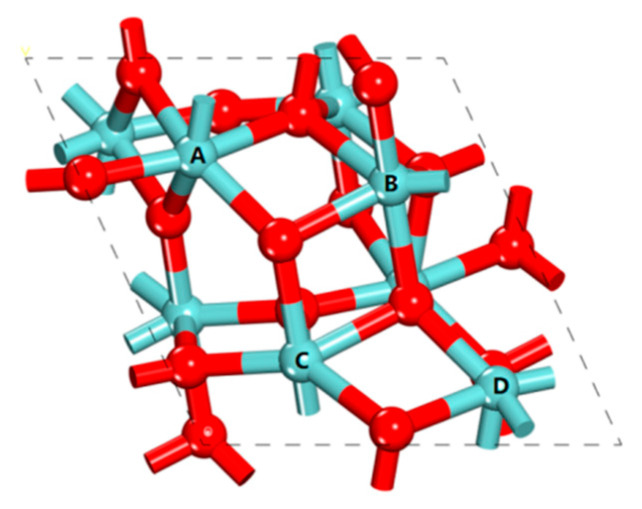
Four surface Zr atoms (**A**, **B**, **C** and **D**) with different coordination environments on the monoclinic ZrO_2_ (111) surface.

**Figure 3:**
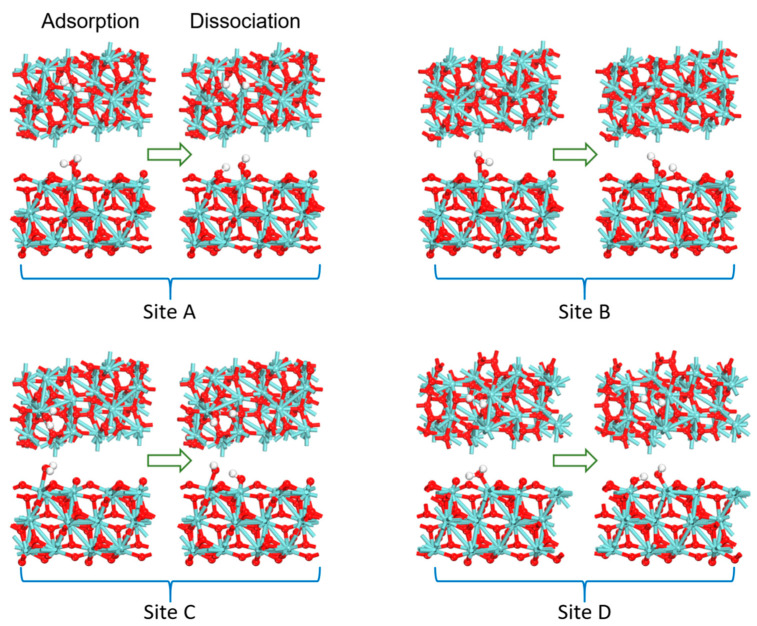
Stable adsorption structures of a water molecule at **A**, **B**, **C** and **D** sites on the monoclinic ZrO_2_ (111) surface and the corresponding dissociation structures.

As shown in Figure 2, Zr atoms of monoclinic ZrO_2_ bulk have seven-fold coordination with O atoms and O atoms have three- or four-fold coordination with Zr atoms. Due to the difference in the relative spatial positions of O atoms coordinated to Zr atoms, there are four nonequivalent Zr atoms with six-fold coordination on the surface of monoclinic ZrO_2_ (111). In other words, there are four Lewis acid active sites of surface Zr atoms with different coordination environments on the monoclinic ZrO_2_ (111) surface, labeled **A**, **B**, **C** and **D**, respectively. Considering the four orientations of a water molecule at a site, 16 initial structures of a water molecule adsorbed on the surface were investigated. The most stable adsorption structures at the four sites are shown in Figure 3. The corresponding adsorption energies were −20.8, −18.6, −19.5 and −34.0 kcal/mol, respectively.

Table 2 lists the bond lengths and bond angles of the four stable adsorption structures. At site **A**, the distances between O and H in the water molecule after adsorption are 0.979 and 1.004 Å, and ∠HOH is about 104.4°, whereas the bond length of H–O in the isolated water molecule is 0.97 Å and ∠HOH is about 103.6°. The distance between the Zr atom at site **A** and the O atom in the water molecule is 2.364 Å. There is an interaction between the Zr atom on the surface and the O atom in the water molecule. Similar trends were observed in the variation of structural parameters at the **B**, **C** and **D**. In these adsorption structures, the structure of the water molecule is distorted in value by about 5%. The distances between the Zr atom on the surface and the water molecules are all about 2.4 Å. All of these indicate that there are strong interactions between the Zr atoms on the surface and the O atoms in the water molecules.

Based on the four most stable adsorption structures, the dissociation states of the water molecule on the ZrO_2_ surface were also investigated. As shown in Figure 3, the water molecule can be dissociated into a H atom and the hydroxyl group, where the hydroxyl group is adsorbed on the Zr atom on the surface and the H atom interacts with the O atom on the surface to form a new hydroxyl group. Table 3 lists the bond lengths and bond angles of the dissociated structures. At the **A** site, after the dissociation of the water molecule, the distances of O and H in the water molecule are 0.972 and 1.642 Å, and the ∠HOH is about 109.9°. The distance between the O atom of the water molecule and the Zr atom on the surface decreases from 2.364 Å to 2.080 Å. Similarly, at the **B** (0.969/1.691 Å and 136.5°, 2.085 Å), **C** (0.973/1.774 Å and 106.7°, 2.073 Å) and **D** (0.972/1.465 Å and 110.8°, 2.091 Å) sites, the water molecules are dissociated. The O atom in the water molecule and the Zr atom on the surface are closer together. The distances between Zr and O atoms are below 2.1 Å. All these indicate that the water molecule is dissociated on the ZrO_2_ surface and a new Zr–O bond is formed.

**Figure 4:**
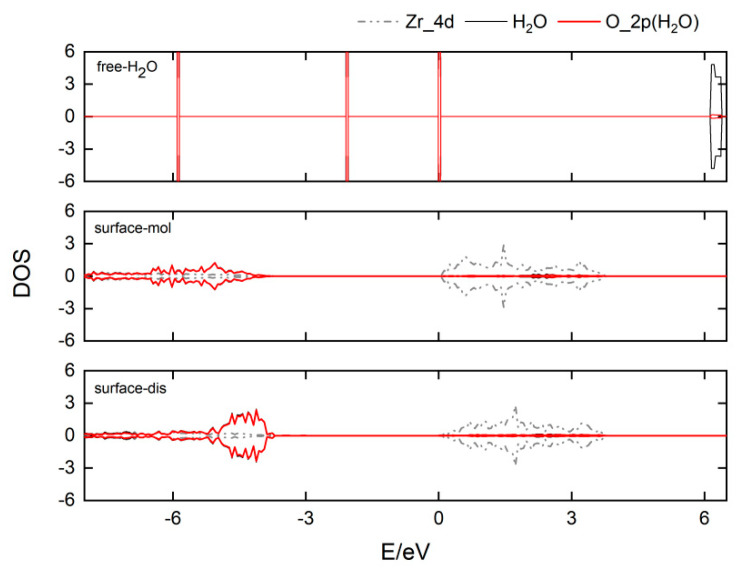
Density of states (DOS) of H_2_O adsorbed on ZrO_2_ (111) surfaces and the corresponding dissociative state.

For comparison, it can be seen that the adsorption energies of the water molecule at the four sites are −20.8, −18.6, −19.5 and −34.0 kcal/mol, respectively. The energies of the dissociated structures of the water molecules are −19.3, −16.5, −12.3 and −33.9 kcal/mol, respectively. These indicate that the adsorption energy and stability of the adsorbed structure are almost the same as that of the dissociated structure at the four sites. Among them, the energy of the dissociation structure at the **C** site is higher by 6.8 kcal/mol than that of the adsorption structure. This is because the different coordination environments of Zr atoms on the surface affect the adsorption and dissociation of small molecules. At the **D** site, the adsorption energy of water molecules is the largest, indicating that the adsorption structure at the **D** site is the most stable. The bonding characteristics between the water molecule and Zr atom at the **D** site on the substrate were further analyzed. The changes in the projected DOS of adsorbed water and dissociation of a water molecule on the **D** site are shown in Figure 4. The adsorption of a water molecule on the surface of ZrO_2_ (111) is mainly through the interaction between the O atom in the water molecule and the Zr atom on the surface. The highest occupied molecular orbital of the water molecule is mainly contributed by the 2*p* orbital of the O atom and the lowest unoccupied molecular orbital of the O atom is mainly contributed by the 2*s* orbital of the O atom. The electrons of the *d* orbitals of Zr atoms on the surface fill the nonbonding orbital of the O atom. From Figure 4, it can be seen that the DOS of the water molecule is shifted from lower to higher energy levels when the adsorbed state changes to the dissociated state. These are also in accordance with the results of the adsorption and dissociation of water on the HfO_2_ surface [35]. This shows that the water molecule gets more charge in the adsorbed state than in the dissociated adsorbed state. Moreover, the peak of the Zr atom at the Fermi energy level becomes lower in the dissociated state, indicating that the electrons are transferred from the surface to the water molecule.

### 3.3. The Water–Solid Interface Reaction of ZrO_2_

**Figure 5:**
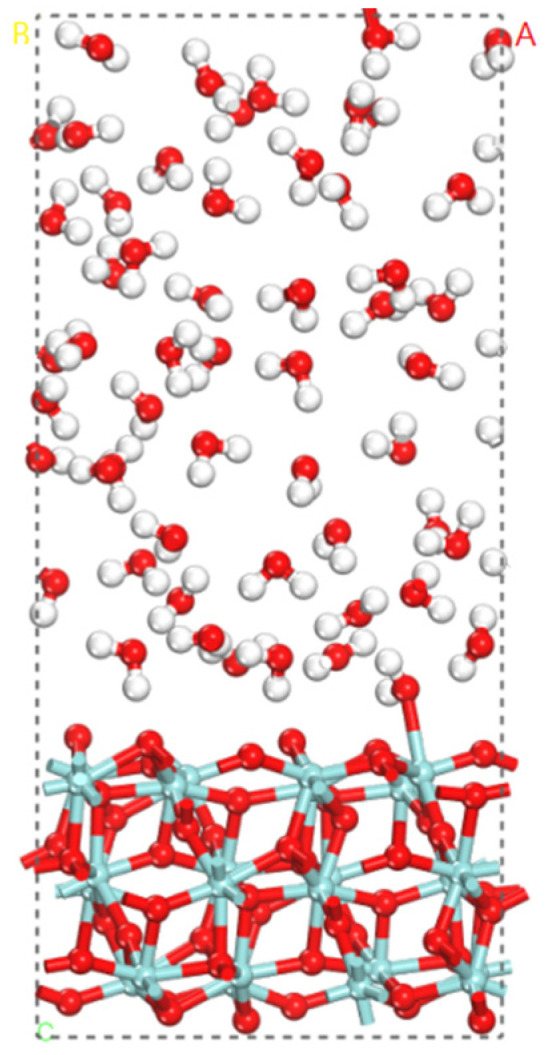
The three-layer model structure of the water–solid interface reactions of ZrO_2_.

To further investigate the adsorption and dissociation behaviors of a large number of water molecules on the ZrO_2_ surface, FPMD simulations of the water–solid interface of ZrO_2_ were conducted at 373 K. Figure 5 shows the three-layer model structure of the water–solid interface of ZrO_2_, in which 55 water molecules are added into the vacuum slab as the explicit solvent corresponding to the density (1 g/mL) of the water.

**Figure 6:**
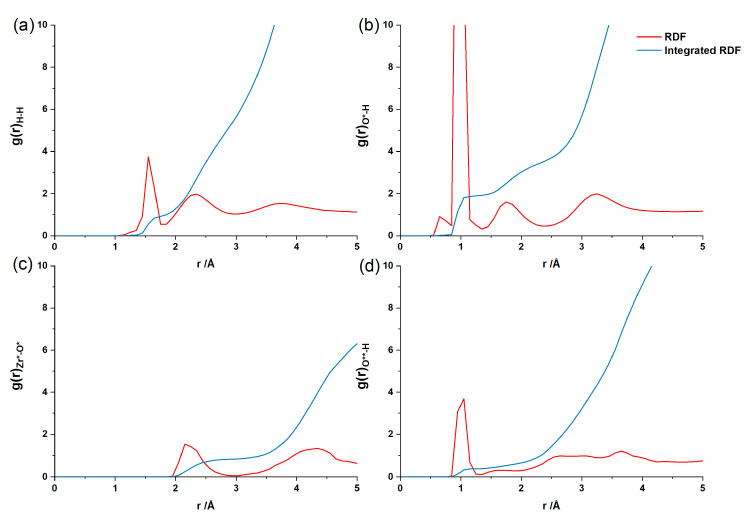
Radial distribution functions (RDFs) and their integrated RDFs. g(*r*)_H–H_ between H atoms in the water molecule (**a**), g(*r*)_O*–H_ between O atoms in the water molecule and H atoms in the water molecule (**b**), g(*r*)_Zr*–O*_ between Zr atoms on the surface of ZrO_2_ and O atoms in the water molecule (**c**) and g(*r*)_O**–H_ between O atoms on the surface of ZrO_2_ and H atoms in the water molecule (**d**).

To understand the distribution and movement rules of the particles on the surface, the radial distribution functions (RDFs) and their integrated RDFs for H and O in water molecules and for the Zr atom on the ZrO_2_ surface were calculated. The RDF g(*r*) is the distribution probability of a given particle *α* over a particle *β* when the distance is *r*. As shown in Figure 6, the first peak in g(*r*)_H–H_ appears around 1.5 Å, showing H–H interactions between the same water molecules. The second peak in g(*r*)_H–H_ appears around 2.3 Å, showing the interaction between H atoms of different water molecules. The first peak in g(*r*)_O*–H_ is 1.0 Å and corresponds to the O–H bond. The second peak in g(*r*)_O*–H_ appears at 1.6–2.1 Å, which shows strong hydrogen bonding between the H and O atoms in different water molecules. The first peak of g(*r*)_Zr*–O*_ is 2.15 Å and corresponds to the interaction between the Zr atom on the surface and the O atom in a water molecule, which also indicates that the water molecules are strongly adsorbed and dissociated on the ZrO_2_ surface. The value of the integral RDF corresponding to the first peak of g(*r*)_Zr*–O*_ is 0.9, indicating that the first coordination layer of the Zr atom on the surface has 0.9 O atoms and the Zr atom has approximately one water molecule adsorbed on it. The peak of g(*r*)_O**–H_ between O atoms on the surface of ZrO_2_ and H atoms in the water molecule is about 1.0 Å and further indicates hydroxyl (–OH) formation on the ZrO_2_ surface. The value of the integrated RDF corresponding to the first peak of g(*r*)_O**–H_ indicates that the first coordination layer of the O atom has 0.4 H atoms on the surface. All these results above show that the water molecules mainly interact with each other via hydrogen bonding. The water molecules close to the surface will be strongly adsorbed at the Zr atoms on the surface to form Zr–O bonding sites. Meanwhile, the H atoms of some water molecules are dissociated to form O–H bonds with the O atoms on the surface.

**Figure 7:**
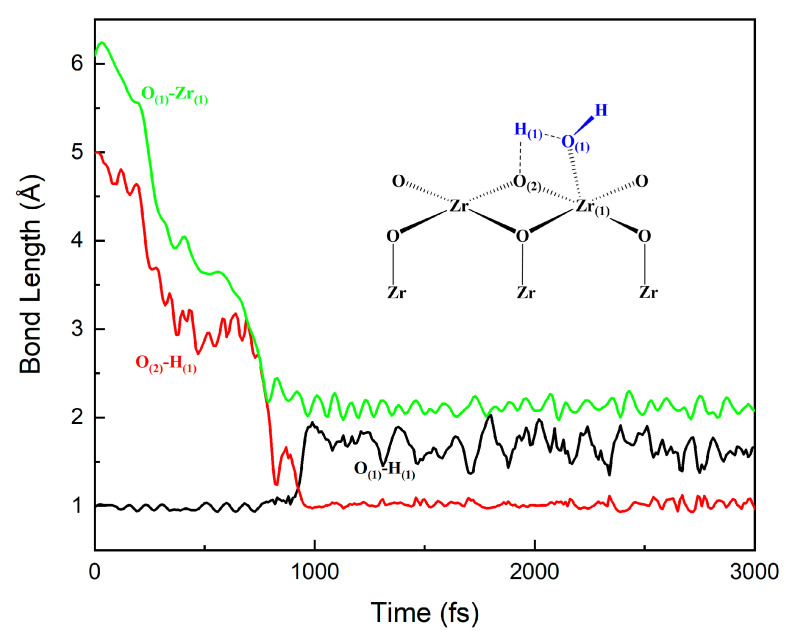
Evolutions of O–H and Zr–O bonds of the interface reaction between the first layer of water and the ZrO_2_ surface at site **A**.

**Figure 8:**
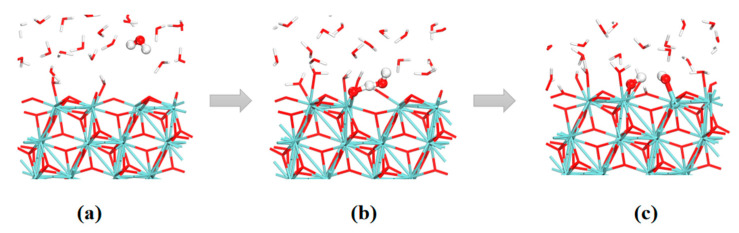
FPMD simulation snapshots at 0 (**a**), 1000 (**b**) and 3000 fs (**c**) of the water–solid interface reaction of ZrO_2_ at site **A**.

**Figure 9:**
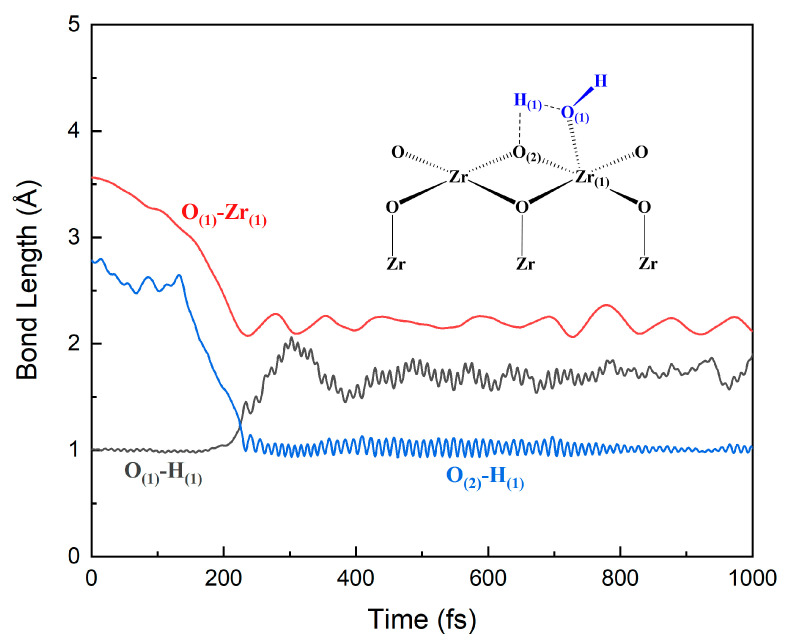
Evolutions of O–H and Zr–O bonds in the interface reaction between the first layer of water and the ZrO_2_ surface with the five-layer ZrO_2_ model.

The adsorption and dissociation behaviors of H_2_O molecules on the ZrO_2_ surface can be obtained by performing FPMD simulations of the water–solid interface of ZrO_2_. Figure 7 and Figure 8 show the evolutions of O–H and Zr–O bonds of the interface reaction between the first layer of water and the ZrO_2_ surface, which is based on the four-membered ring (**4MR**) pathway. Initially, at the surface **A** site, the distance between the O_(1)_ atom in the water molecule and the Zr_(1)_ atom on the surface is around 6.1 Å. With the evolution of the FPMD simulation, the water molecules can go closer to the surface. At about 1000 fs, the distance between the Zr_(1)_ and O_(1)_ atoms decreases to around 2.141 Å, which implies the formation of Zr–O bonds. Similarly, the distance between the O_(1)_ atom and the H_(1)_ atom in the water molecule increases from 1.0 Å to 1.7 Å and the distance between the H_(1)_ atom and the O_(2)_ atom on the ZrO_2_ substrate decreases from 5.0 Å to about 1.0 Å. All these indicate the breakage of the O–H bond in the water molecule and the formation of the H–O bond between the H atom in the water molecule and the O atom on the substrate surface. The distance between the O_(1)_ atom and the H_(1)_ atom remains around 1.7 Å, indicating that strong hydrogen bonds still exist between them. To verify the validity of the three-layer ZrO_2_ model, we also performed FPMD simulations with a five-layer ZrO_2_ surface model, shown in Figure 9 and Figure S1. The simulation time of the FPMD was 1 ps. From Figure 9 and Figure S1, we obtained similar behavior of the water–solid interface between three-layer and five-layer ZrO_2_ surface models.

**Figure 10:**
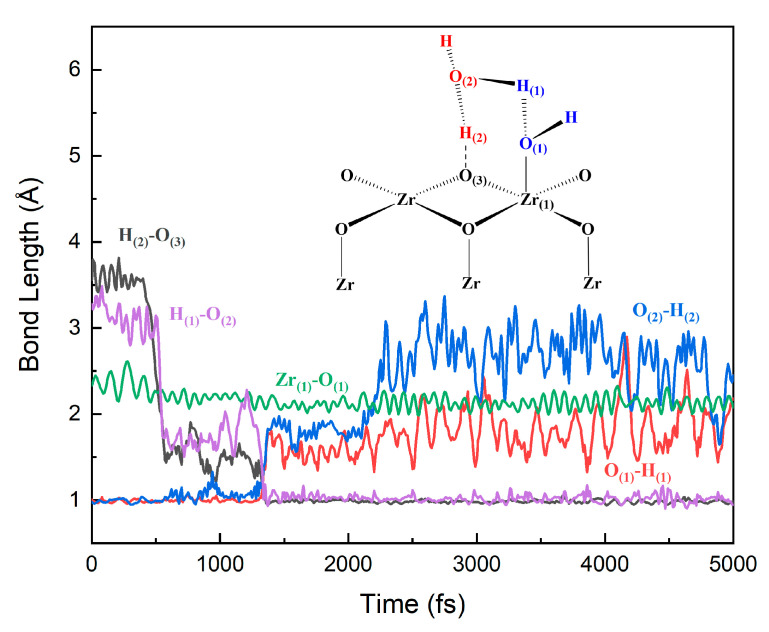
Evolutions of the bond lengths of O–H and Zr–O of two water molecules and the ZrO_2_ interface reaction at site **C**.

**Figure 11:**
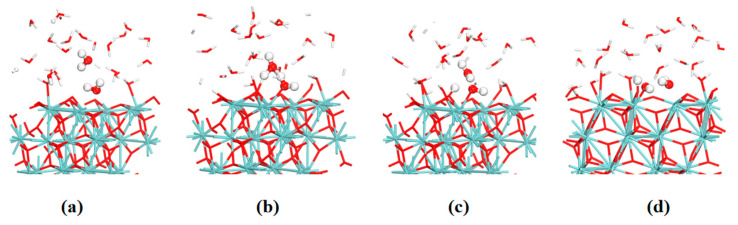
FPMD simulation snapshots at 0 (**a**), 500 (**b**), 1400 (**c**) and 2200 fs (**d**) of the water–solid interface reaction of ZrO_2_ at site **C**.

In addition to the dissociation of the first layer of water, the second layer of water on the surface also participates in the interface reaction. This water–solid interface reaction is based on the six-membered ring (**6MR**) pathway. Figure 10 and Figure 11 show the evolutions of the bond lengths of O–H and Zr–O bonds at the reaction center. The whole reaction process involves the ZrO_2_ surface at site **C** and two water molecules, H_2_O(a)(H_(1)_O_(1)_H) and H_2_O(b)(H_(2)_O_(2)_H), via a **6MR** pathway. In the beginning, the distances of the O_(1)_ atom and H_(1)_ atom, the H_(2)_ atom and the O_(2)_ atom are both 1.0 Å. The distances between the H_(1)_ atom and O_(2)_ atom, and H_(2)_ and O_(3)_ atom are 3.3 and 3.7 Å, respectively. The distance between Zr_(1)_ and O_(1)_ atoms is about 2.5 Å. At 500 fs, the H_(1)_–O_(2)_ and H_(2)_–O_(3)_ distances gradually become shorter and their distances both decrease to about 1.6 Å. The Zr_(1)_–O_(1)_ distance also decreases to 2.1 Å. All these indicate that H_(1)_–O_(2)_, H_(2)_–O_(3)_ and Zr_(1)_–O_(1)_ bonds have the tendency to form. At 1400 fs, the H_(1)_–O_(2)_ and H_(2)_–O_(3)_ distances decrease to 1.0 Å. In contrast, the O_(1)_–H_(1)_ and O_(2)_–H_(2)_ distances increase to 1.7 Å. Afterward, the lengths of O_(1)_–H_(1)_ and O_(2)_–H_(2)_ bonds oscillate between 1.5 and 2.5 Å. At 2200 fs, the O_(1)_–H_(1)_ and O_(2)_–H_(2)_ distances oscillate around 3.0 Å. The distance between O_(2)_ and H_(1)_ is reduced to about 1.0 Å.

All these show that the H_2_O (a) molecule can be dissociated to form the hydroxyl group, which can be strongly adsorbed to the Zr_(1)_ atom on the substrate surface. This whole process of distance change shows that the dissociated H_(2)_ atom in H_2_O (b) dissociated from the H_2_O (b) molecule can form a H_(2)_–O_(3)_ bond with the O_(3)_ atom on the surface. The remaining hydroxyl portion of the H_2_O (b) molecule can combine with the dissociated H_(1)_ atom in H_2_O (a) to form a new water molecule. DFT calculations and FPMD simulation results show that water molecules can not only be dissociated directly on the ZrO_2_ surface but also exchange the proton with the help of adjacent water and finally be dissociated.

## 4. Conclusions

In summary, the adsorption and dissociation behaviors of water molecules on the surface of monoclinic ZrO_2_ (111) were investigated using DFT static calculations and FPMD simulations. Four Lewis acid active sites of surface Zr atoms with different coordination environments have different adsorption and dissociation states and energies of water molecule on the ZrO_2_ (111) surface. DOS analysis shows that the O atom of the water molecule prefers to bond with the Zr atom on the ZrO_2_ (111) surface. There are strong interactions between the *p* orbitals of the O atom and the *d* orbitals of the Zr atom. The electrons of the *d* orbitals of the Zr atom on the surface fill the nonbonding orbitals of O atoms. H atoms of the water molecule prefer to bond with O atoms on the surface. Through FPMD simulations, the water–solid interface reaction of the ZrO_2_ (111) surface was analyzed. The water molecules mainly interact with each other by hydrogen bonding. Through the 4MR or 6MR pathways of water–solid interface reactions, the first and second water molecules on the surface can be adsorbed and dissociated at the Zr sites on the surface to form Zr–O and O–H bonds. These insights can provide the theoretical significance for the adsorption and dissociation of water and the water–solid interface behavior of metal oxides, such as H_2_O-based ALD.

## Figures and Tables

**Table 1:** Structural parameters and adsorption energies of a water molecule on different models of the ZrO_2_ surface.

Layer	*d*_O–Zr_/(Å)	*d*_O–H_/(Å)	∠HOH/(°)	*E*_ads_/(kcal/mol)
2	2.418	0.976/0.994	103.6	−18.3
3	2.404	0.976/0.995	103.6	−18.5
4	2.402	0.977/0.993	103.8	−18.7

**Table 2:** Structural parameters of water adsorption on ZrO_2_ surfaces.

Site	*d*_O–Zr_/(Å)	*d*_O–H_/(Å)	∠HOH/(°)	*E*_ads_/(kcal/mol)
**A**	2.364	0.979/1.004	104.4	−20.8
**B**	2.394	0.974/0.998	107.8	−18.6
**C**	2.354	0.977/1.000	106.4	−19.5
**D**	2.234	0.974/1.067	107.9	−34.0

**Table 3:** Structural parameters and related energies of water dissociation on ZrO_2_ surfaces.

Site	*d*_O–Zr_/(Å)	*d*_O–H_/(Å)	∠HOH/(°)	*E*_ads_/(kcal/mol)
**A**	2.080	0.972/1.642	109.9	−19.3
**B**	2.085	0.969/1.691	136.5	−16.5
**C**	2.073	0.973/1.774	106.7	−12.3
**D**	2.091	0.972/1.465	110.8	−33.9

## Supplementary Materials

The following supporting information can be downloaded at: https://www.mdpi.com/article/10.3390/nano12244362/s1, Figure S1: Radial distribution functions (RDFs) and their integrated RDFs of the interface reaction between the first layer water and ZrO2 surface on the five-layer ZrO2 model.; Table S1: Atomic coordinates of 2-layer ZrO2 substrate; Table S2: Atomic coordinates of 3-layer ZrO2 substrate; Table S3: Atomic coordinates of 4-layer ZrO2 substrate; Table S4: Atomic coordinates of 5-layer ZrO2 substrate; Table S5: Atomic coordinates of 3-layer ZrO2 substrate 55 waters.
