# Supplementary material for: First-Principles Molecular Dynamics Simulations on Water–Solid Interface Behavior of H2O-Based Atomic Layer Deposition of Zirconium Dioxide
Source: Nanomaterials (Basel). 2022 Dec 7;12(24):4362. doi: 10.3390/nano12244362 (PMC9783483; doi:10.3390/nano12244362)
Supplement: Supplementary file 1 [file nanomaterials-12-04362-s001.zip › nanomaterials-2064189-supplementary.pdf]

Supplementary Material

First-Principles Molecular Dynamics Simulations on Water–Solid Interface Behavior of H_2O-Based Atomic Layer Deposition of Zirconium Dioxide

Rui Xu [1], Zhongchao Zhou [1], Yingying Wang [1], Hongping Xiao [1], Lina Xu [1,*], Yihong Ding [1], Xinhua Li [1], Aidong Li [2] and Guoyong Fang [1,*]

[1] Key Laboratory of Carbon Materials of Zhejiang Province, College of Chemistry and Materials Engineering, Wenzhou University, Wenzhou 325035, China
[2] National Laboratory of Solid State Microstructures, College of Engineering and Applied Sciences, Nanjing University, Nanjing 210093, China
* Correspondence: xulina@wzu.edu.cn (L.X.); fanggy@wzu.edu.cn (G.F.)

Content

Figure S1 Radial distribution functions (RDFs) and their integrated RDFs of the interface reaction between the first layer water and ZrO_2 surface on the five-layer ZrO_2 model.

Table S1 Atomic coordinates of 2-layer ZrO_2 substrate.

Table S2 Atomic coordinates of 3-layer ZrO_2 substrate.

Table S3 Atomic coordinates of 4-layer ZrO_2 substrate.

Table S4 Atomic coordinates of 5-layer ZrO_2 substrate.

Table S5 Atomic coordinates of 3-layer ZrO_2 substrate 55 waters.

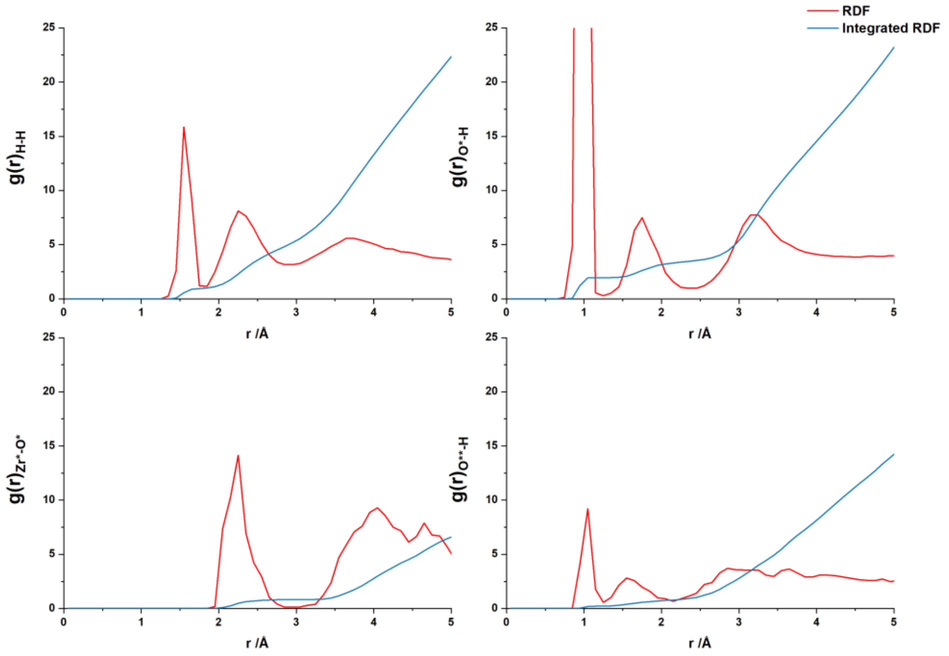

Figure S1 Radial distribution functions (RDFs) and their integrated RDFs of five-layer ZrO_2 model. $g(r)_{H\text{-}H}$ between H atoms in the water molecule (a), $g(r)_{O^*\text{-}H}$ between O atoms in the water molecule and H atoms in the water molecule (b),

$g(\mathrm{r})_{\mathrm{Zr^{*}\text{-}O^{*}}}$ between Zr atoms on the surface of ZrO_2 and O atoms in the water molecule (c) and $g(\mathrm{r})_{\mathrm{O^{**}\text{-}H}}$ between O atoms on the surface of ZrO_2 and H atoms in the water molecule (d).

Table S1 Atomic coordinates of 2-layer ZrO_2 substrate

ZrO2_2		
1.0		
13.3698997498	0.0000000000	0.0000000000
0.7368347366	7.4012122236	0.0000000000
0.0000000000	0.0000000000	25.2518005371
Zr	O	
16	32	
Cartesian		
6.053911234	6.539044964	2.005498007
3.096552434	3.010285094	4.919045587
12.370443741	2.838438852	2.005498007
10.149919655	6.710890985	4.919045587
1.706319432	0.684094068	2.096152038
8.759686471	4.384700125	2.096152038
6.026418488	0.854646041	4.983791156
13.079785742	4.555252263	4.983791156
2.241506500	4.066891920	1.862825364
8.558038826	0.366286001	1.862825364
6.371726887	4.076581264	4.683281741
12.688259363	0.375974849	4.683281741
5.852137069	2.520704760	1.772171333

3.868424212	6.374288169	4.573114522
12.905504312	6.221310871	1.772171333
10.184956143	2.673682278	4.573114522
4.107730155	6.911160524	2.518243348
1.287448534	3.354980965	5.626581709
10.424263082	3.210554633	2.518243348
8.340815577	7.055587077	5.626581709
6.604547215	4.496015976	2.373695885
3.938707508	0.897409140	5.382616815
12.921079340	0.795410030	2.373695885
10.992074795	4.598015693	5.382616815
2.452574104	2.284845275	3.127773094
9.505941702	5.985450946	3.127773094
6.671597166	2.512946976	5.899967649
13.724963989	6.213552867	5.899967649
6.506656467	0.630672866	2.951299868
4.438999778	4.571057223	5.631052681
13.560023716	4.331279033	2.951299868
10.755532273	0.870451001	5.631052681
4.086715020	3.429277777	1.233550454
1.360709830	0.088043520	4.139301865
11.140082640	7.129883668	1.233550454

8.414077580	3.788649714	4.139301865
1.695480609	6.102817564	1.434302280
8.012013016	2.402211453	1.434302280
5.937466176	6.193024550	4.223421774
12.253998262	2.492418218	4.223421774
5.202830262	0.878967943	0.727756906
2.876785068	4.749263696	3.698377118
12.256197499	4.579573999	0.727756906
9.193317386	1.048657694	3.698377118
0.948947184	2.532324782	0.986840401
8.002314007	6.232930674	0.986840401
5.238656712	2.674876241	3.775167447
12.292024000	6.375482794	3.775167447

Table S2 Atomic coordinates of 3-layer ZrO_2 substrate

```
ZrO2_3
1.0
        13.3698997498         0.0000000000         0.0000000000
         0.7368347366         7.4012122236         0.0000000000
         0.0000000000         0.0000000000        28.0905990601
   Zr      O
   24     48
```

Cartesian

1.765640884	6.373775998	1.987128961
8.082172992	2.673169886	1.987128961
6.031256346	6.482460410	4.880540198
3.110545299	3.016356154	7.738651114
12.347788454	2.781854298	4.880540198
10.163912963	6.716962486	7.738651114
4.471550399	4.219431159	2.077861541
1.669172845	0.671688804	4.986156109
10.788082900	0.518824992	2.077861541
8.722539607	4.372295081	4.986156109
6.003352368	0.846997495	7.790594696
13.056719617	4.547603662	7.790594696
4.269768863	0.201016924	1.844709599
2.223774448	4.055170654	4.684262657
11.323136098	3.901622953	1.844709599
8.540306581	0.354564790	4.684262657
6.352721322	4.056400570	7.535910658
12.669253015	0.355794293	7.535910658
1.564000499	2.355435794	1.753696150
8.617367443	6.056041905	1.753696150
5.813455977	2.458469117	4.562973342

3.852796072	6.388742779	7.474131223
12.866823220	6.159075229	4.562973342
10.169328601	2.688136888	7.474131223
6.142545646	3.083359047	2.447554063
4.122488931	6.986168244	5.512046893
1.308331149	3.395897976	8.416951570
13.195912889	6.783965159	2.447554063
10.439022213	3.285561912	5.512046893
8.361698271	7.096503868	8.416951570
2.301238706	4.345528510	2.366958924
8.617771423	0.644922508	2.366958924
6.618483374	4.446725781	5.295693715
3.923722515	0.910921627	8.228526637
12.935015897	0.746119835	5.295693715
10.977089786	4.611528014	8.228526637
5.191962766	5.808395865	3.132608977
2.455323485	2.304018831	5.908833007
11.508494875	2.107789754	3.132608977
9.508690529	6.004624943	5.908833007
6.583127589	2.478678926	8.758032719
13.636494433	6.179285038	8.758032719
2.233092328	0.454498287	2.943793716

9.286459579	4.155104481	2.943793716
6.529637024	0.649096693	5.723015844
4.404096228	4.545164563	8.483525449
13.583003853	4.349702639	5.723015844
10.720629521	0.844558341	8.483525449
6.851945771	6.964614702	1.215199333
4.109452707	3.555745912	4.038023531
1.339700249	0.059917320	7.109912505
13.168478278	3.264008590	1.215199333
11.162820348	7.256352024	4.038023531
8.393068100	3.760523529	7.109912505
3.723876546	2.236942487	1.416047063
1.599627624	6.057151389	4.211030813
10.777244187	5.937548598	1.416047063
7.916160109	2.356545057	4.211030813
5.961208345	6.180087925	7.134455674
12.277740453	2.479481813	7.134455674
0.914559906	0.713698922	0.709287651
7.967927547	4.414305033	0.709287651
5.024953489	0.834997480	3.666380833
2.928349074	4.757210952	6.471500036
12.078321131	4.535603592	3.666380833

9.244881193	1.056604951	6.471500036
3.714044254	6.067661708	0.968563906
0.971345470	2.513473264	3.841023098
10.030576561	2.367055596	0.968563906
8.024712641	6.214079155	3.841023098
5.205057283	2.662667288	6.584494719
12.258424548	6.363273620	6.584494719

Table S3 Atomic coordinates of 4-layer ZrO_2 substrate.

```
ZrO2_4
1.0
      13.3698997498         0.0000000000         0.0000000000
       0.7368347366         7.4012122236         0.0000000000
       0.0000000000         0.0000000000        30.9293994904
   Zr    O
   32   64
Direct
     0.265089989         0.338849992         0.065130003
     0.081281401         0.858739555         0.158556074
     0.765089989         0.838850021         0.065130003
     0.581281364         0.358739555         0.158556074
     0.401672155         0.876958013         0.251208812
```

0.208299726	0.408661872	0.344038367
0.901672184	0.376957983	0.251208812
0.708299696	0.908661842	0.344038367
0.483520001	0.047770001	0.068070002
0.300120145	0.569904685	0.160696134
0.117677435	0.089774728	0.254667014
0.983519971	0.547770023	0.068070002
0.800120115	0.069904670	0.160696134
0.617677391	0.589774728	0.254667014
0.440553159	0.116057396	0.345856994
0.940553188	0.616057396	0.345856994
0.498349994	0.504830003	0.060520001
0.315760911	0.024372872	0.152600586
0.135021374	0.545625091	0.245221913
0.998350024	0.004830000	0.060520001
0.815760911	0.524372876	0.152600586
0.635021389	0.045625135	0.245221913
0.443273097	0.549162984	0.337672293
0.943273067	0.049162939	0.337672293
0.279929996	0.795920014	0.057580002
0.097221680	0.312522948	0.150195941
0.779929996	0.295920014	0.057580002

0.597221673	0.812522948	0.150195941
0.415301174	0.332732171	0.241803318
0.239375845	0.863570452	0.335084498
0.915301204	0.832732141	0.241803318
0.739375830	0.363570452	0.335084498
0.117613547	0.399083465	0.081092745
0.617613554	0.899083495	0.081092745
0.437042534	0.432707906	0.177992851
0.255664051	0.947919488	0.271293253
0.070826821	0.461379290	0.366075248
0.937042534	0.932707906	0.177992851
0.755664051	0.447919518	0.271293253
0.570826828	0.961379230	0.366075248
0.320426673	0.065154560	0.077727646
0.139899984	0.583789587	0.171892449
0.820426643	0.565154552	0.077727646
0.639900029	0.083789580	0.171892449
0.459420592	0.602360964	0.265259653
0.284524441	0.123598337	0.359894753
0.959420562	0.102360994	0.265259653
0.784524441	0.623598397	0.359894753
0.023950234	0.764072001	0.102276556

0.523950279	0.264071971	0.102276556
0.342372090	0.788417697	0.193574309
0.161505893	0.310324073	0.285537183
0.842372060	0.288417697	0.193574309
0.661505878	0.810324013	0.285537183
0.472524375	0.336301833	0.377257884
0.972524345	0.836301804	0.377257884
0.344549149	0.539035082	0.095374949
0.164805099	0.066873610	0.186910331
0.844549119	0.039035074	0.095374949
0.664805055	0.566873610	0.186910331
0.481123894	0.087432139	0.278835684
0.293816805	0.616187394	0.368200660
0.981123865	0.587432146	0.278835684
0.793816805	0.116187401	0.368200660
0.141120002	0.918680012	0.040169999
0.641120017	0.418680012	0.040169999
0.460611045	0.954544604	0.131906807
0.277938187	0.469387949	0.225353017
0.097995251	0.005423663	0.322837710
0.960611045	0.454544604	0.131906807
0.777938187	0.969387949	0.225353017

0.597995281	0.505423665	0.322837710
0.442360014	0.779910028	0.046659999
0.255310744	0.295898587	0.137492880
0.076060325	0.818808079	0.231054395
0.942359984	0.279909998	0.046659999
0.755310774	0.795898557	0.137492880
0.576060295	0.318808079	0.231054395
0.398113191	0.836350739	0.324313402
0.898113191	0.336350739	0.324313402
0.243579999	0.574100018	0.023820000
0.053226847	0.094299652	0.118925810
0.743579984	0.074100003	0.023820000
0.553226888	0.594299674	0.118925810
0.373929530	0.112163015	0.210060567
0.183955207	0.642224967	0.302669376
0.873929560	0.612163007	0.210060567
0.683955193	0.142224997	0.302669376
0.413100004	0.297490001	0.032200001
0.235297740	0.818150938	0.124789290
0.050721988	0.337317616	0.217489138
0.913100004	0.797490001	0.032200001
0.735297740	0.318150938	0.124789290

0.550721943	0.837317586	0.217489138
0.367773026	0.360264391	0.307359070
0.867772996	0.860264421	0.307359070

Table S4 Atomic coordinates of 5-layer ZrO_2 substrate.

ZrO2_5		
1.0		
13.3698997498	0.0000000000	0.0000000000
0.7368347366	7.4012122236	0.0000000000
0.0000000000	0.0000000000	33.7681999207
Zr	O	
40	80	
Direct		
0.445589989	0.816519976	0.037840001
0.265089989	0.338849992	0.121909998
0.084600002	0.861180007	0.205970004
0.945590019	0.316520005	0.037840001
0.765089989	0.838850021	0.121909998
0.584599972	0.361180007	0.205970004
0.404110014	0.883509994	0.290039986
0.223619998	0.405849993	0.374110013
0.904110014	0.383509994	0.290039986

0.723619998	0.905849993	0.374110013
0.164010003	0.025440000	0.040530000
0.664009988	0.525439978	0.040530000
0.483520001	0.047770001	0.124600001
0.303030014	0.570100009	0.208660007
0.122529998	0.092430003	0.292730004
0.983519971	0.547770023	0.124600001
0.803030014	0.070100002	0.208660007
0.622529984	0.592429996	0.292730004
0.442039996	0.114759997	0.376800001
0.942040026	0.614759982	0.376800001
0.178849995	0.482499987	0.033620000
0.678849995	0.982500017	0.033620000
0.498349994	0.504830003	0.117689997
0.317860007	0.027160000	0.201749995
0.137370005	0.549489975	0.285820007
0.998350024	0.004830000	0.117689997
0.817860007	0.527159989	0.201749995
0.637369990	0.049490001	0.285820007
0.456880003	0.571820021	0.369890004
0.956879973	0.071819998	0.369890004
0.460420012	0.273589998	0.030929999

0.279929996	0.795920014	0.115000002
0.099440001	0.318250000	0.199059993
0.960420012	0.773590028	0.030929999
0.779929996	0.295920014	0.115000002
0.599439979	0.818250000	0.199059993
0.418940008	0.340579987	0.283129990
0.238450006	0.862909973	0.367199987
0.918940008	0.840579987	0.283129990
0.738449991	0.362910002	0.367199987
0.302819997	0.902670026	0.056480002
0.122330002	0.425000012	0.140540004
0.802820027	0.402669996	0.056480002
0.622330010	0.925000012	0.140540004
0.441839993	0.447329998	0.224610001
0.261339992	0.969669998	0.308679998
0.080849998	0.492000014	0.392749995
0.941839993	0.947329998	0.224610001
0.761340022	0.469669998	0.308679998
0.580850005	0.991999984	0.392749995
0.001580000	0.041439999	0.050530002
0.501580000	0.541440010	0.050530002
0.321090013	0.063770004	0.134599999

0.140599996	0.586109996	0.218669996
0.821089983	0.563769996	0.134599999
0.640600026	0.086110003	0.218669996
0.460099995	0.608439982	0.302740008
0.279610008	0.130769998	0.386799991
0.960099995	0.108439997	0.302740008
0.779609978	0.630770028	0.386799991
0.200360000	0.247250006	0.071460001
0.019859999	0.769580007	0.155520007
0.700360000	0.747250021	0.071460001
0.519860029	0.269580007	0.155520007
0.339370012	0.791909993	0.239590004
0.158879995	0.314240009	0.323659986
0.839370012	0.291909993	0.239590004
0.658879995	0.814239979	0.323659986
0.478390008	0.336580008	0.407730013
0.978389978	0.836579978	0.407730013
0.030840000	0.523859978	0.063780002
0.530839980	0.023860000	0.063780002
0.350340009	0.546190023	0.147850007
0.169850007	0.068520002	0.231920004
0.850340009	0.046190001	0.147850007

0.669849992	0.568520010	0.231920004
0.489360005	0.090860002	0.315979987
0.308869988	0.613189995	0.400050014
0.989359975	0.590860009	0.315979987
0.808870018	0.113190003	0.400050014
0.321610004	0.396349996	0.014980000
0.141120002	0.918680012	0.099050000
0.821609974	0.896350026	0.014980000
0.641120017	0.418680012	0.099050000
0.460630000	0.941009998	0.183119997
0.280129999	0.463340014	0.267179996
0.099639997	0.985669971	0.351249993
0.960630000	0.441009998	0.183119997
0.780130029	0.963339984	0.267179996
0.599640012	0.485670000	0.351249993
0.122850001	0.757579982	0.020919999
0.622850001	0.257580012	0.020919999
0.442360014	0.779910028	0.104989998
0.261869997	0.302240014	0.189060003
0.081370004	0.824570000	0.273130000
0.942359984	0.279909998	0.104989998
0.761870027	0.802240014	0.189060003

0.581369996	0.324570000	0.273130000
0.400880009	0.846899986	0.357190013
0.900879979	0.346899986	0.357190013
0.424080014	0.051770002	0.000000000
0.243579999	0.574100018	0.084069997
0.063089997	0.096430004	0.168139994
0.924080014	0.551769972	0.000000000
0.743579984	0.074100003	0.084069997
0.563090026	0.596430004	0.168139994
0.382600009	0.118759997	0.252200007
0.202110007	0.641089976	0.336270005
0.882600009	0.618759990	0.252200007
0.702109993	0.141090006	0.336270005
0.093599997	0.275160015	0.007680000
0.593599975	0.775160015	0.007680000
0.413100004	0.297490001	0.091739997
0.232610002	0.819819987	0.175809994
0.052120000	0.342150003	0.259880006
0.913100004	0.797490001	0.091739997
0.732609987	0.319819987	0.175809994
0.552119970	0.842149973	0.259880006
0.371630013	0.364479989	0.343950003

0.871630013	0.864480019	0.343950003

Table S5 Atomic coordinates of 3-layer ZrO_2 substrate 55 waters.

ZrO2_3_H2O		
1.0		
13.3849000931	0.0000000000	0.0000000000
0.3679334631	7.4097707366	0.0000000000
0.0000000000	0.0000000000	29.3668003082
O H Zr		
103 110 24		
Direct		
0.269927055	0.249232650	0.147246912
0.575825512	0.477526098	0.503127337
0.526521206	0.738670707	0.437544078
0.980632067	0.719191074	0.118247636
0.113621846	0.813443482	0.422109663
0.621419907	0.096191898	0.589543521
0.695391536	0.596496582	0.107669577
0.599495173	0.579717517	0.206534564
0.384490699	0.939312696	0.583393335
0.164949924	0.023006292	0.198039785
0.606706679	0.962872386	0.163158953

0.260983795	0.747459888	0.237141311
0.874694765	0.580532610	0.493748695
0.564058006	0.689767957	0.346103460
0.874921501	0.214320123	0.508036435
0.065945826	0.362076819	0.142482489
0.851144016	0.294450909	0.238863379
0.552332699	0.946769834	0.270393789
0.201030359	0.254914939	0.266140103
0.392943203	0.184508204	0.284445882
0.945271313	0.279057860	0.322249770
0.291288853	0.531355977	0.554732144
0.693829715	0.649717271	0.566670775
0.323916644	0.866639495	0.319780201
0.985788345	0.513978660	0.417426407
0.548580110	0.776864290	0.622021317
0.382330418	0.538095951	0.183631465
0.739869893	0.881065667	0.028500812
0.442848265	0.042747293	0.113463528
0.101161666	0.548304915	0.225744933
0.831039190	0.842400789	0.240890205
0.915922046	0.255281776	0.062340252
0.060298737	0.583289146	0.314350843

0.517997921	0.001574615	0.506330311
0.250526428	0.882603168	0.500046790
0.427831411	0.285391659	0.598716855
0.035166282	0.173593611	0.565871298
0.193017289	0.239394635	0.515910625
0.179474100	0.755340517	0.611307085
0.214778587	0.481116831	0.373444647
0.725579917	0.138559803	0.104590066
0.834165871	0.165477097	0.419135064
0.112360269	0.228882983	0.431920469
0.808745205	0.960566044	0.565632701
0.394136995	0.506777287	0.333230317
0.832512856	0.482830226	0.621314228
0.724566281	0.101359069	0.289821506
0.969254196	0.698529422	0.579547107
0.381120205	0.693103552	0.095216714
0.523349702	0.445007503	0.084474631
0.967168808	0.983071864	0.186177447
0.012998209	0.926356316	0.341063380
0.326287806	0.830970883	0.412753254
0.816101789	0.892054617	0.357297152
0.746417224	0.595487833	0.317837596

0.437314957	0.437445581	0.766051352
0.257683724	0.979646444	0.872204602
0.071901515	0.505962133	0.969227791
0.937946081	0.940918922	0.766866744
0.758771181	0.476100922	0.871039689
0.565810919	0.002886477	0.969389737
0.133346736	0.594106317	0.763573527
0.633022785	0.093548141	0.763306379
0.457538873	0.609882116	0.863204777
0.280356050	0.129758984	0.963602483
0.958613276	0.111578815	0.863564670
0.784696817	0.624612093	0.962228954
0.332936972	0.789538383	0.792100251
0.146460906	0.330309302	0.887791574
0.836864114	0.291799784	0.791589320
0.648081958	0.828685701	0.888577521
0.457780033	0.359480619	0.982963383
0.949378490	0.859735608	0.985812902
0.166819811	0.076216996	0.784422100
0.665485740	0.577271044	0.784216523
0.479824364	0.102793008	0.877569735
0.290079653	0.632174373	0.973226368

0.982282221	0.604304433	0.877169132
0.791856885	0.140015602	0.970598459
0.462104321	0.940535545	0.722162068
0.273796052	0.460728347	0.818445563
0.093286484	0.997488201	0.925237060
0.963588953	0.443551660	0.721037507
0.773914993	0.966481745	0.819444060
0.594383538	0.503398776	0.924292862
0.249186516	0.312099636	0.726949215
0.074927077	0.831537545	0.827678084
0.751326144	0.811824679	0.728099525
0.573786795	0.330130696	0.826952696
0.398014635	0.847777128	0.927107871
0.901787817	0.350172013	0.926289499
0.072389327	0.082680151	0.707659304
0.571430862	0.588018715	0.707175434
0.384198487	0.110485360	0.803403914
0.200554788	0.650514901	0.897992969
0.884801447	0.614075720	0.803070724
0.692609966	0.152300477	0.899538159
0.240944758	0.810110331	0.717070937
0.051749285	0.338997483	0.812985539

0.741209507	0.312543303	0.718626976
0.550836504	0.839594483	0.813736320
0.364388704	0.366396934	0.906201363
0.867656052	0.867187977	0.906703591
0.307105571	0.333959699	0.166083083
0.240090698	0.160193697	0.168178916
0.588475704	0.347671360	0.500608087
0.624380350	0.523718059	0.525173485
0.543388903	0.630716562	0.456614733
0.532744825	0.841041684	0.459051907
0.000864288	0.594414532	0.126405895
0.937722564	0.709037840	0.091779090
0.072891735	0.705550790	0.421731710
0.180640638	0.768311620	0.411966473
0.693430901	0.064580649	0.585446358
0.598501384	0.997707307	0.608966827
0.675357103	0.670558274	0.134086639
0.712622225	0.681260467	0.083699368
0.654337466	0.511901557	0.221625283
0.598544061	0.699418783	0.220512047
0.314223766	0.912530839	0.587091982
0.390595496	0.071333565	0.589849591

0.092827156	0.993880749	0.195796803
0.203316748	0.914672971	0.210526049
0.597382486	0.981041253	0.195871741
0.540252507	0.995425701	0.148479953
0.316830754	0.676120162	0.222078204
0.279288679	0.781861067	0.268747717
0.906563461	0.563928723	0.463238657
0.809046924	0.639493346	0.488571137
0.562120795	0.727463424	0.378675699
0.509781420	0.603146434	0.344297856
0.865250766	0.347856104	0.507762849
0.865110278	0.179039672	0.475369930
0.138591304	0.329555035	0.138457090
0.038685858	0.255451113	0.156428695
0.797841668	0.222319797	0.252035528
0.882052898	0.218476191	0.214970544
0.546051562	0.862716496	0.296324342
0.493079156	0.033425659	0.272703260
0.181031331	0.164752066	0.243247420
0.170537949	0.368321627	0.253590614
0.322888196	0.215276152	0.273054004
0.407188058	0.294357210	0.302113742

0.990007520	0.381275594	0.320708334
0.914723516	0.281262606	0.291117847
0.303755581	0.631702840	0.534071207
0.252031416	0.593203068	0.579477727
0.635583162	0.691861808	0.586346209
0.729879975	0.760449827	0.560528815
0.314340413	0.868691504	0.353344887
0.351352870	0.984592259	0.311862826
0.027563527	0.398426920	0.422058374
0.964760303	0.513608217	0.385617107
0.542604268	0.702444494	0.649657369
0.480472952	0.816044629	0.610701621
0.454108685	0.524375021	0.190946683
0.378498822	0.600053072	0.153665274
0.723107338	0.991187453	0.046690326
0.813297868	0.880820155	0.024232833
0.421362936	0.923215985	0.103481412
0.381550699	0.108979374	0.124466062
0.083629720	0.501310825	0.195554957
0.159375161	0.626318455	0.223619685
0.791262627	0.936055422	0.258341640
0.824907720	0.731088400	0.258133978

0.966910660	0.284588188	0.084588237
0.863115489	0.201270431	0.080234610
0.067198299	0.567922533	0.280844033
0.042406108	0.713708639	0.318696111
0.456121266	0.969116211	0.521612644
0.561843514	0.026904928	0.532455325
0.187063217	0.851270556	0.486280978
0.239281878	0.010383335	0.509854436
0.392857581	0.380103499	0.580160141
0.498200029	0.277989239	0.589469790
0.978748918	0.188939661	0.543808639
0.016385637	0.248002231	0.592060089
0.230358541	0.342502117	0.527127385
0.135603920	0.225368619	0.537527204
0.178727955	0.781845272	0.643929243
0.109087065	0.750607252	0.601711512
0.191586211	0.371120989	0.387656450
0.165263310	0.512703776	0.349189758
0.689993024	0.074220225	0.129325986
0.713779986	0.266492605	0.111473665
0.818211973	0.054546591	0.402461410
0.866279840	0.238550350	0.396271229

0.100040980	0.100334741	0.429061443
0.144282058	0.241104648	0.462724566
0.870421648	0.892989278	0.575363934
0.830189586	0.054326482	0.543859005
0.333890736	0.482759088	0.352340668
0.374646276	0.626099467	0.320116282
0.822464943	0.355408728	0.626228929
0.775107920	0.528677642	0.601887345
0.752744675	0.039521705	0.317464858
0.656680286	0.054130726	0.284184366
0.930326939	0.609129548	0.596268833
0.957994998	0.666782439	0.547508359
0.325359493	0.674737632	0.074715339
0.433160454	0.593762636	0.087635212
0.589976609	0.485224158	0.093840763
0.523084342	0.313946843	0.083074532
0.964162886	0.900217533	0.159800276
0.924433410	0.923953116	0.209371567
0.053861734	0.914571464	0.369048446
0.006412951	0.055627346	0.331826329
0.306951672	0.879520774	0.443099529
0.393694341	0.774140596	0.418454468

0.889166355	0.892786622	0.350487918
0.794818521	0.771083593	0.347343951
0.675200522	0.613601863	0.328948915
0.765750468	0.468342155	0.322020710
0.092075042	0.879552424	0.752866864
0.592658699	0.380290836	0.751721740
0.399645329	0.883744478	0.850381732
0.204078212	0.429316700	0.947021902
0.901593626	0.384870350	0.849885702
0.700042248	0.932367086	0.949630439
0.295509994	0.562106073	0.752752364
0.118204147	0.101606742	0.853855789
0.795309365	0.066164173	0.753843307
0.618635833	0.602246642	0.854060709
0.433737040	0.137555867	0.947558522
0.936665714	0.635679245	0.947500467
0.329120547	0.011211901	0.743853867
0.132706285	0.558031738	0.840509653
0.830273926	0.515113652	0.742794454
0.632086694	0.057926230	0.841936171
0.440312803	0.562227190	0.938551486
0.938710988	0.064494662	0.940100133

0.096256152	0.305469930	0.743159950
0.597720504	0.809032440	0.744357884
0.413747638	0.339140296	0.837144434
0.236358479	0.876348913	0.938130856
0.914188206	0.841014981	0.837629914
0.739043057	0.379238099	0.937455773
